# Mechanical analysis of skier activity in giant slalom

**DOI:** 10.1371/journal.pone.0347192

**Published:** 2026-04-28

**Authors:** Jurij Hladnik, Radovan Dražumerič, Igor Petrović, Ivan Bon, Mateja Očić, Vjekoslav Cigrovski, Boris Jerman

**Affiliations:** 1 Department of Engineering Design and Transportation Systems, Faculty of Mechanical Engineering, University of Ljubljana, Ljubljana, Slovenia; 2 Department for Management of Production Technologies, Faculty of Mechanical Engineering, University of Ljubljana, Ljubljana, Slovenia; 3 Laboratory for aeronautics, Faculty of Mechanical Engineering, University of Ljubljana, Ljubljana, Slovenia; 4 Department of Kinesiology of Sports, Faculty of Kinesiology, University of Zagreb, Zagreb, Croatia; 5 Laboratory for Sports Games, Faculty of Kinesiology, University of Zagreb, Zagreb, Croatia; University of Innsbruck, AUSTRIA

## Abstract

Propulsive mechanical work in alpine skiing can be performed by gravity and by the skier’s activity. In this study, the part of the work performed by the skier’s activity for the movement of the skier’s centre of mass (COM) relative to the skis (i.e., external skier activity mechanical work, $W_{SA}$) was analysed. The main objectives of the study were to determine (a) positive and negative $W_{SA}$, (b) $W_{SA}$ performed in the skiing direction ($W_{SA,t}$), and (c) mechanical work performed by individual external forces in giant slalom, including the corresponding power profiles. For this purpose, three-dimensional whole-body kinematics of five skilled skiers were measured with a global navigation satellite system and an inertial motion capture system, while skiing on a predefined giant slalom course. $W_{SA}$ was calculated upon a two-point skier model as the difference between the work performed by the ground reaction force (GRF) at the COM and at the midpoint between the ankle joints. The negative $W_{SA}$ was 3.1 times larger than the positive $W_{SA}$, which answers an important question about the dominant muscle activity in alpine skiing. $W_{SA,t}$ was exclusively positive on the expense of negative $W_{SA}$ performed in other directions. $W_{SA,t}$ and its power are a characteristic of ski turn mechanics and only partly reflect the skier’s own propulsion. $W_{SA,t}$ presented 2.4%, gravity 93.1% and GRF 4.5% of the total positive work in the skiing direction, respectively. Regarding energy dissipation, 81.0% of the total negative work was dissipated in the ski-snow contact, 14.6% against the air drag and 4.4% was absorbed in the skiers’ bodies. Moreover, a new parameter for evaluation of skiing propulsion efficiency, considering also skier activity mechanical work, was defined.

## Introduction

Both professional and recreational athletes, coaches and researchers are interested in the physiological and biomechanical parameters of athletes during exercise. In addition to heart rate and speed of movement, they are also interested in mechanical power and the associated mechanical work performed by the athlete’s muscles, i.e., with their activity. In terrestrial locomotion, such as walking [1,2], running [1–5], roller skiing [6–9] and cycling [10], numerous studies have been conducted to evaluate the mechanical work that athletes put into their movement.

In human locomotion on the flat and uphill, the athlete is the only motor that moves the body [11], therefore the total propulsive mechanical work corresponds to the mechanical work produced by the athlete’s activity. In downhill locomotion, such as alpine skiing, gravity is the main driving force. In order to obtain the mechanical work resulting from the skier’s activity (i.e., skier activity mechanical work, SAMW), the gravity mechanical work needs to be excluded.

Although alpine skiing is mostly powered by gravity, it can be physically demanding, especially in competitions, where success is highly dependent on the skier’s effort [12]. The SAMW is performed for the movement of the skier’s centre of mass (COM) relative to the skis, for pole and ski push-offs (e.g., skating), and for the movement of the skier’s body segments relative to the COM. The COM movements relative to the skis, and pole and ski push-offs, require external forces, therefore the associated work belongs to the external SAMW ($W_{SA}$), while the segmental movements require forces internal to the body, therefore the associated work belongs to the internal SAMW [1].

Meyer [11] evaluated $W_{SA}$ in giant slalom by summing the products between the ground reaction forces (GRFs) and the skier’s COM displacements relative to the midpoint between the skier’s feet in the mediolateral, longitudinal and anteroposterior directions. The GRFs were obtained from the skier’s COM kinematics with addition of the centrifugal force, which was already implicitly considered in the GRFs. Thus, the average $W_{SA}$ calculated over the ski turns was slightly above zero, implying that the positive and negative $W_{SA}$ were almost equal, although other researchers [13,14] speculated that $W_{SA}$ should be mostly negative.

To explain how skiers can propel themselves without pole and skating ski push-offs during a ski turn or on an undulating snow surface, the pumping mechanism in skiing was studied [15–17]. According to Lind and Sanders [16], skiers can increase their kinetic rotational energy during a ski turn by shortening the radius of the axis around which they rotate. This can be achieved by moving their COM closer to the ski turn centre. The increase in kinetic rotational energy is supposed to be proportional to the work done against the centrifugal force. Thus, a larger extension of the skier’s body leads to a larger increase in the kinetic rotational energy [18]. Magelssen et al [19] experimentally demonstrated that skiers improved their race times when performing the pumping strategy in slalom skiing, as the skiing times when descending a slalom course were shorter, than when skiing in a straight line between the same starting and end points.

Since the existing studies [11] on the external SAMW done for the movements of the skier’s COM relative to the skis ($W_{SA}$) are insufficient to a certain extent, the main objective of the present study was to introduce a two-point model of the skier, which would enable the evaluation of the a) positive and negative $W_{SA}$, b) $W_{SA}$ performed in the skiing direction ($W_{SA,t}$), and c) the mechanical work performed by individual external forces in giant slalom, including the corresponding power profiles within a ski turn.

## Materials and methods

### Experimental setup

The study involved five certified alpine ski instructors (age 32.7 ± 12.7 years, weight 76.5 ± 12.1 kg, height 178 ± 7 cm). The subjects were informed about the study procedure before the measurements and gave their written consent for participation. The study was conducted in accordance with the Helsinki Declaration and was approved by the Ethics Committee of the Faculty of Kinesiology at the University of Zagreb, Croatia.

**Fig 1:**
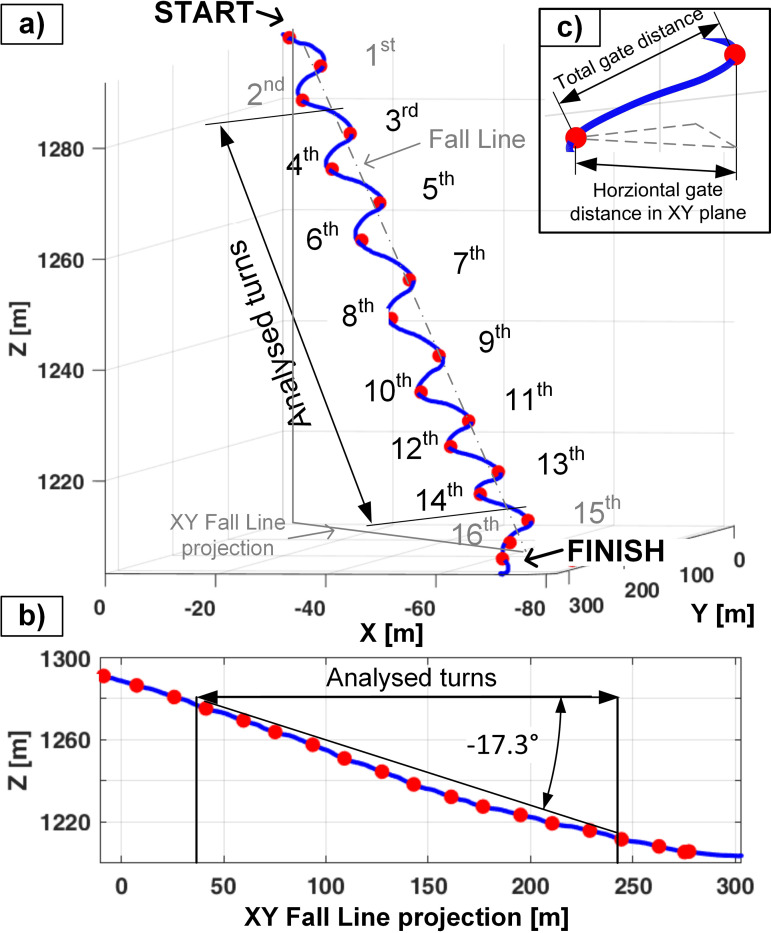
a) Skiing trajectory of a random run, b) profile of the trajectory and c) gate distance definitions. Blue line presents the skiing trajectory. The start, the finish and the gates are denoted as red dots.

The measurements were taken of four consecutive days from 24^th^ till 27^th^ of February 2025. Each subject performed three runs on a predefined ski slope with sixteen similar giant slalom turns (Fig 1a). For the analysis, only the measurements taken from the 3^rd^ to the 14^th^ turn of each run were taken (six left and six right turns). The total distance between the analysed gates was 19.1 ± 0.6 m, the horizontal distance (transversal to the fall line, Fig 1c) was 5.8 ± 0.6 m, and the inclination between the gates was −16.4 ± 2.5°. The ski slope was in the shade all day and had an average incline of −17.3° (Fig 1b).

The measurements were taken on four consecutive days with air temperature between −1℃ and 2℃. The snow on the slope was compact. To ensure comparable conditions between runs, the ski course was smoothed by course slippers and groomed at the end of each day.

### Instruments

Except for the helmet, the skiers used their own skiing equipment. Their full-body kinematics was measured by the Xsens MVN Link inertial motion capture system (Xsens Technologies B.V., Enschede, The Netherlands; sampling frequency 240 Hz, mass 1.9 kg) consisting of 17 miniature sensors with three-dimensional accelerometers, gyroscopes and magnetometers distributed over the body.

**Fig 2:**
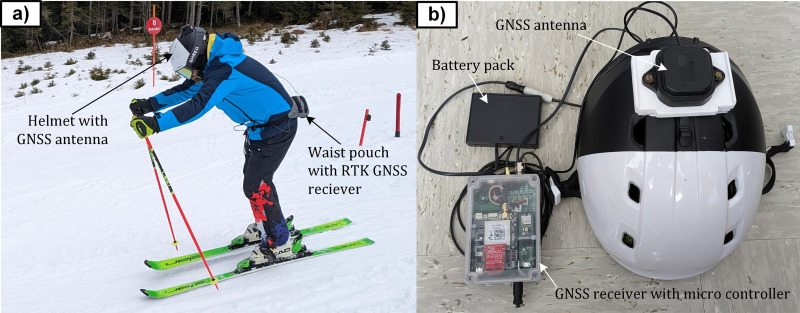
a) A skier during the measurements. b) The custom-built Real-Time Kinematics Global Navigation Satellite System (RTK GNSS) including helmet.

The global position of the skiers was measured using a custom-built Real-Time Kinematics Global Navigation Satellite System (RTK GNSS), which enabled sub-centimetre accuracy and a sampling frequency of 100 Hz (Fig 2). The system consisted of a multiband GNSS antenna (ANN-MB-00, u-blox AG, Thalwil, Switzerland) mounted on the skier’s helmet and connected to a high-precision GNSS receiver (simpleRTK3B Pro, based on the mosaic-X5 module, Septentrio NV, Leuven, Belgium). The receiver was interfaced with a microcontroller (Teensy 4.1, PJRC, Sherwood, Oregon, USA) responsible for logging GPS and GLONASS signals using both L1 and L2 frequency bands. The entire setup, including rechargeable batteries, weighed 700 g and was carried in a waist pouch secured around the skier’s hips (Fig 2a). This system was also used to set up the gate positions. Combination of inertial motion capture systems and RTK GNSSs is a well-established method that has been used in previous alpine skiing studies [20,21,22].

### Skier model

In the MVN BIOMECH software (Xsens, MVN Studio 4.4, firmware version 4.3.1) each skier’s body was simplified as a linked segment model consisting of 15 rigid segments: head, torso, pelvis, two upper arms, two forearms, two hands, two thighs, two shanks and two feet. The mass-dimension characteristics of the body segments were obtained from the anthropometric models [23,24] according to the height, mass and sex of the skier. The mass of the clothing, of the measuring systems and of the helmet were added to the mass of the skier and were thus evenly distributed over the model. The mass of the skis was assumed to be proportional to the length of the skis multiplied by $\text{2}.\text{1}\text{8}\;[kg\cdot m^{-\text{1}}]$, while the mass of the ski boots was assumed to be proportional to the skier’s mass multiplied by  0.029 [/]. These masses were added to the masses of the skier’s feet. The mass of the ski poles was taken the same for all skiers (0.246 *kg*) and was added to the mass of the skier’s hands. As an output of the inertial motion capture system, the joint positions and the position of the skier’s COM were obtained.

### Data processing

The data from both measurement systems were processed using MATLAB R2024b (MathWorks, Natick, MA, United States). The raw GNSS data, originally expressed in geographic coordinates (latitude and longitude in decimal degrees), were first converted to UTM zone 33N (WGS 84) coordinates using a publicly available MATLAB function  [25]. The GNSS data were then resampled to 240 Hz using cubic spline interpolation to match the sampling frequency of the inertial motion capture system.

The data were aligned in the same global coordinate system (X pointing East, Y North and Z up) and manually synchronised to the same event, i.e., head movement at the start. Since the joint and COM positions of the skiers were given in a local coordinate system, they had to be transformed into the global coordinate system by considering the global and local position of the GNSS antenna as described in [20]. The global positions (trajectories) were filtered using a second-order zero-phase digital Butterworth low-pass filter with a normalized cut-off frequency of 0.017.

**Fig 3:**
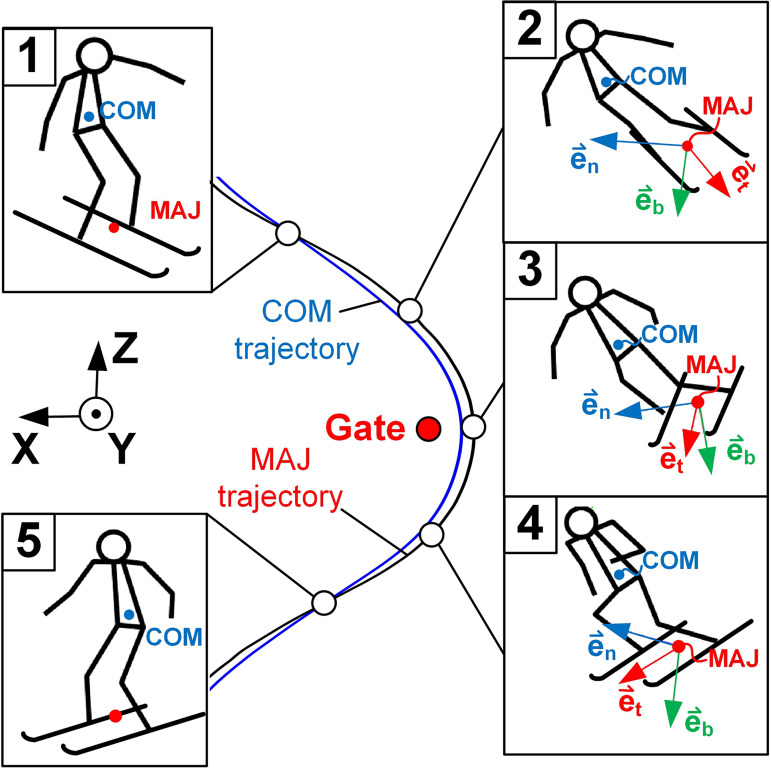
Skier positions along a ski turn including the trajectories of his centre of mass (COM) and of the midpoint between the ankle joints (MAJ). In the skier’s positions within the ski turn (positions from 2 to 4) also the tangential $\backslash ({\overset{⇀}{e}}_{t}$, normal $\backslash ({\overset{⇀}{e}}_{n}$ and binormal $\backslash ({\overset{⇀}{e}}_{b}$ unit vectors of the MAJ trajectory are annotated.

The global trajectories (Fig 3) of the skier’s COM and of the GRF application point, approximated by the midpoint between the ankle joints (MAJ), were the main input data for the power-work calculations. The velocities of the skier’s COM and MAJ, $\backslash ({\overset{⇀}{v}}_{COM}$ and $\backslash ({\overset{⇀}{v}}_{MAJ}$, and the accelerations, $\backslash ({\overset{⇀}{a}}_{COM}$ and $\backslash ({\overset{⇀}{a}}_{MAJ}$, were calculated as first and second order derivatives of the COM and MAJ trajectories with respect to time. The tangential $\backslash ({\overset{⇀}{e}}_{t}$, normal $\backslash ({\overset{⇀}{e}}_{n}$ and binormal $\backslash ({\overset{⇀}{e}}_{b}$ unit vectors of the MAJ trajectories, which were also required for the power-work calculations, were calculated using the Frenet–Serret formulae [26], where $\backslash ({\overset{⇀}{e}}_{t}$ was oriented in the skiing direction, $\backslash ({\overset{⇀}{e}}_{n}$ in the direction of the ski turn centre and $\backslash ({\overset{⇀}{e}}_{b}$ perpendicular to the plane formed by ${\overset{⇀}{e}}_{t}$ and $\backslash ({\overset{⇀}{e}}_{n}$.

### Power-work calculations

In skiing, the skier is loaded with gravity ${\overset{⇀}{F}}_{g}$, air drag ${\overset{⇀}{F}}_{d}$ and ground reaction ${\overset{⇀}{F}}_{GRF}$ external forces [27]. In order to turn, the skier leans toward the ski turn centre and ${\overset{⇀}{F}}_{GRF}$ increases in the ${\overset{⇀}{e}}_{n}$ direction, which forces the skier to turn (Fig 3, pos.2).

**Fig 4:**
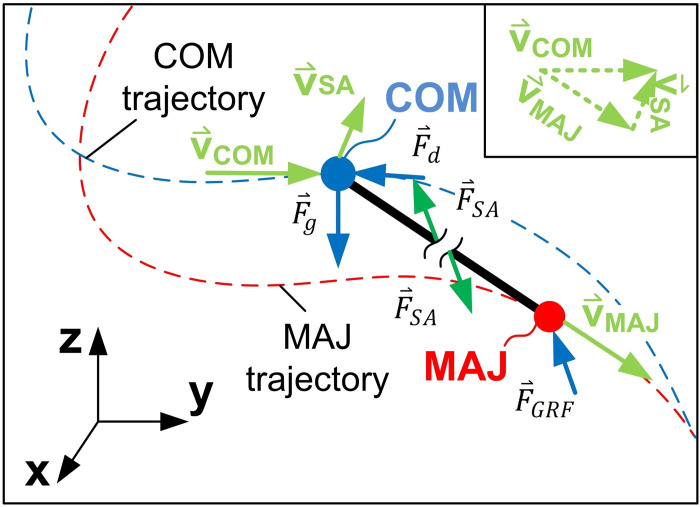
Two-point skier model including the trajectories of the centre of mass (COM) and of the midpoint of the ankle joints (MAJ). $\backslash ({\overset{⇀}{v}}_{COM}$ and $\backslash ({\overset{⇀}{v}}_{MAJ}$ represent the velocity vectors of the COM and of the MAJ, while $\backslash ({\overset{⇀}{v}}_{SA}$ presents the velocity vector of the COM relative to the MAJ. ${\overset{⇀}{𝐅}}_{g}$ is the gravity, ${\overset{⇀}{𝐅}}_{d}$ the air drag and ${\overset{⇀}{𝐅}}_{GRF}$ the ground reaction force vector. ${\overset{⇀}{𝐅}}_{SA}$ is the skier activity force transmitted by between the MAJ and the COM.


To calculate $W_{SA}$ and its power, a two-point model of the skier was used, consisting of two coupled points, where the upper point is a mass point, representing the skier’s COM, and the lower point is the MAJ (Fig 4). To calculate the skier activity force ${\overset{⇀}{F}}_{SA}$ between the skier’s MAJ and the COM, the two-point skier model was imaginarily divided into two parts. The dynamic force equilibrium at the COM was written as follows:

$${\overset{⇀}{F}}_{g}+{\overset{⇀}{F}}_{d}+{\overset{⇀}{F}}_{SA}=m\cdot ({\overset{⇀}{a}}_{COM}$$ (1)

and the dynamic force equilibrium at the MAJ as:


$${\overset{⇀}{F}}_{GRF}-{\overset{⇀}{F}}_{SA}=\text{0}$$ (2)

Here, ${\overset{⇀}{F}}_{g}$ was calculated as the product between the gravitational acceleration vector $\overset{⇀}{g}$ and the mass of the skier *m* including clothes and equipment.

The drag force vector was calculated using the Rayleigh drag equation as follows:


$${\overset{⇀}{F}}_{d}=-\rho \cdot S\cdot C_{d}\cdot \frac{{v_{COM}}^{\text{2}}}{\text{2}}{\cdot \overset{⇀}{e}}_{t}$$ (3)

where ρ is the air density, S is the cross-sectional area of the skier and $C_{d}$ is the coefficient of aerodynamic drag. The product $S\cdot C_{d}$ was obtained from the regression model for the tuck skier position as follows [28]:


$$S\cdot C_{d}=\;\text{0}.\text{5}\text{8}\text{4}\cdot H_{n}-\text{0}.\text{0}\text{4}\text{5}$$ (4)

where $H_{n}$ is the instantaneous mid-shoulder height, normalised to the skier’s height, both measured from the snow surface.

From the force equilibrium of the lower part of the skier’s model (Equation 2), ${\overset{⇀}{F}}_{SA}$ equals the external force ${\overset{⇀}{F}}_{GRF}$, therefore according to work definitions in biomechanics [1], ${\overset{⇀}{F}}_{SA}$ produces external mechanical work.

By multiplying the force equilibrium at the skier’s COM (Equation 1) with ${\overset{⇀}{v}}_{COM}$ and by considering the equality of ${\overset{⇀}{F}}_{SA}$ and ${\overset{⇀}{F}}_{GRF}$ (Equation 2), the power balance at the COM may be derived:


$$\left({\overset{⇀}{F}}_{g}+{\overset{⇀}{F}}_{d}+{\overset{⇀}{F}}_{GRF}\right)\cdot {\overset{⇀}{v}}_{COM}=m\cdot {\overset{⇀}{a}}_{COM}\cdot {\overset{⇀}{v}}_{COM}$$

$$P_{g}+P_{d}+P_{GRF}=P_{inert}$$ (5)

where $P_{g}$, $P_{d}$, $P_{GRF}$ and $P_{inert}$ are the gravitational, air drag, GRF and inertial power at the COM, respectively. By combining the force equilibria of the upper and the lower part of the two-point skier model (Equations 1, 2), and by multiplying them with their corresponding velocities, also the power balance of the two-point skier model can be derived:


$$\left({\overset{⇀}{F}}_{g}+{\overset{⇀}{F}}_{d}\right)\cdot {\overset{⇀}{v}}_{COM}+{\overset{⇀}{F}}_{GRF}\cdot {\overset{⇀}{v}}_{MAJ}+{\overset{⇀}{F}}_{SA}\cdot \left({\overset{⇀}{v}}_{COM}-{\overset{⇀}{v}}_{MAJ}\right)=m\cdot {\overset{⇀}{a}}_{COM}\cdot {\overset{⇀}{v}}_{COM}$$

$$P_{g}+P_{d}+P_{GRF,\;MAJ}+P_{SA}=P_{inert}$$ (6)

where $P_{GRF,MAJ}$ presents the GRF power at the MAJ and $P_{SA}$ the mechanical power of the ${\overset{⇀}{F}}_{SA}$. Since ${\overset{⇀}{F}}_{SA}$ equals ${\overset{⇀}{F}}_{GRF}$ (Equation 2), $P_{SA}$ may be calculated as the difference between the GRF power at the COM and at the MAJ:


$$P_{SA}={\overset{⇀}{F}}_{SA}\cdot \left({\overset{⇀}{v}}_{COM}-{\overset{⇀}{v}}_{MAJ}\right){=\overset{⇀}{F}}_{GRF}\cdot \left({\overset{⇀}{v}}_{COM}-{\overset{⇀}{v}}_{MAJ}\right)=P_{GRF}-P_{GRF,MAJ}$$ (7)

Note, that $P_{SA}$ may also be obtained as:


$$P_{SA}={\overset{⇀}{F}}_{GRF}\cdot {\overset{⇀}{v}}_{SA}$$ (8)

where ${\overset{⇀}{v}}_{SA}$ is the velocity vector of the skier’s COM relative to the MAJ (Fig 4).

In general, power can be written as a sum of components related to the orthogonal coordinate system $\left({\overset{⇀}{e}}_{t},{\overset{⇀}{e}}_{n},{\overset{⇀}{e}}_{b}\right)$:


$$P=\overset{⇀}{F}\cdot \overset{⇀}{v}=\left[\left(\overset{⇀}{F}\cdot {\overset{⇀}{e}}_{t}\right){\overset{⇀}{e}}_{t}+\left(\overset{⇀}{F}\cdot {\overset{⇀}{e}}_{n}\right){\overset{⇀}{e}}_{n}+\left(\overset{⇀}{F}\cdot {\overset{⇀}{e}}_{b}\right){\overset{⇀}{e}}_{b}\right]\cdot \left[\left(\overset{⇀}{v}\cdot {\overset{⇀}{e}}_{t}\right){\overset{⇀}{e}}_{t}+\left(\overset{⇀}{v}\cdot {\overset{⇀}{e}}_{n}\right){\overset{⇀}{e}}_{n}+\left(\overset{⇀}{v}\cdot {\overset{⇀}{e}}_{b}\right){\overset{⇀}{e}}_{b}\right]$$

$$P=\overset{⇀}{F}\cdot \left[\left(\overset{⇀}{v}\cdot {\overset{⇀}{e}}_{t}\right){\overset{⇀}{e}}_{t}+\left(\overset{⇀}{v}\cdot {\overset{⇀}{e}}_{n}\right){\overset{⇀}{e}}_{n}+\left(\overset{⇀}{v}\cdot {\overset{⇀}{e}}_{b}\right){\overset{⇀}{e}}_{b}\right]$$

$$P=P_{t}+P_{n}+P_{b}$$ (9)

Analogously, the mechanical-power balance of the skier’s two-point model (Equation 6) can be written in the tangential, normal and binormal directions of the MAJ local coordinate system:

${\overset{⇀}{F}}_{g}\cdot \left({\overset{⇀}{v}}_{COM}\cdot {\overset{⇀}{e}}_{t}\right){\overset{⇀}{e}}_{t}+{\overset{⇀}{F}}_{d}\cdot {\overset{⇀}{v}}_{COM}+{\overset{⇀}{F}}_{GRF}\cdot {\overset{⇀}{v}}_{MAJ}+{\overset{⇀}{F}}_{SA}\cdot \left[\left({\overset{⇀}{v}}_{COM}\cdot {\overset{⇀}{e}}_{t}\right){\overset{⇀}{e}}_{t}-{\overset{⇀}{v}}_{MAJ}\right]=$


$$=m{\overset{⇀}{a}}_{COM}\cdot \left({\overset{⇀}{v}}_{COM}\cdot {\overset{⇀}{e}}_{t}\right){\overset{⇀}{e}}_{t}$$ (10)

$${\overset{⇀}{F}}_{g}\cdot \left({\overset{⇀}{v}}_{COM}\cdot {\overset{⇀}{e}}_{n}\right){\overset{⇀}{e}}_{n}+{\overset{⇀}{F}}_{SA}\cdot \left({\overset{⇀}{v}}_{COM}\cdot {\overset{⇀}{e}}_{n}\right){\overset{⇀}{e}}_{n}=m{\overset{⇀}{a}}_{COM}\cdot \left({\overset{⇀}{v}}_{COM}\cdot {\overset{⇀}{e}}_{n}\right){\overset{⇀}{e}}_{n}$$ (11)

$${\overset{⇀}{F}}_{g}\cdot \left({\overset{⇀}{v}}_{COM}\cdot {\overset{⇀}{e}}_{b}\right){\overset{⇀}{e}}_{b}+{\overset{⇀}{F}}_{SA}\cdot \left({\overset{⇀}{v}}_{COM}\cdot {\overset{⇀}{e}}_{b}\right){\overset{⇀}{e}}_{b}=m{\overset{⇀}{a}}_{COM}\cdot \left({\overset{⇀}{v}}_{COM}\cdot {\overset{⇀}{e}}_{b}\right){\overset{⇀}{e}}_{b}$$ (12)

where a condensed formulation of these equations yields:


$$P_{g,t}+P_{d}+P_{GRF,MAJ}+P_{SA,t}=P_{inert,t}$$ (13)

$$P_{g,n}+P_{SA,n}=P_{inert,n}$$ (14)

$$P_{g,b}+P_{SA,b}=P_{inert,b}$$ (15)

The direction of ${\overset{⇀}{e}}_{t}$ represents the direction of skiing. In this manner, positive $P_{SA,t}$ presents the power for skier propulsion resulting from the skier activity.

Integration of the power balance equations in the individual directions of the MAJ coordinate system (Equations 13–15) over time results in the following mechanical-work balances:


$$W_{g,t}+W_{d}+W_{GRF}+W_{SA,t}=W_{inert,t}$$ (16)

$$W_{g,n}+W_{SA,n}=W_{inert,n}$$ (17)

$$W_{g,b}+W_{SA,b}=W_{inert,b}$$ (18)

Finally, the summation of the mechanical-work components (Equations 16–18) results in the overall mechanical-work balance of the two-point skier model:


$$W_{g}+W_{d}+W_{GRF}+W_{SA}=W_{inert}$$ (19)

where $W_{g}$ is the total work done by the gravity force, $W_{d}$ the total work done by the air drag, $W_{GRF}$ the total work done by the GRF at the MAJ, $W_{SA}$ the external SAMW and $W_{inert}$ the work done by the inertial forces. Here, $W_{inert}$ equals the change of the skier’s COM kinetic energy, and $W_{g}$ the change of his COM potential energy.

By separating the integrations of the positive and negative power values, Equation 19 may be rewritten in its extended form as:


$$\overset{+}{\underset{g}{W}}+\overset{-}{\underset{g}{W}}+\overset{+}{\underset{d}{W}}+\overset{-}{\underset{d}{W}}+\overset{+}{\underset{GRF}{W}}+\overset{-}{\underset{GRF}{W}}\overset{+}{\underset{inert}{+\overset{+}{\underset{SA}{W}}+\overset{-}{\underset{SA}{W}}=W}}+\overset{-}{\underset{inert}{W}}$$ (20)

where the positive work was marked with a “+” sign and the negative with a “-” sign. This equation states that the work done by the gravity, air drag, GRF and by the skier activity (left side) results in the mechanical work of the inertial forces (right side). In a similar manner, also the work balance in individual directions of the MAJ coordinate system (Equations 16–18) in their extended formulations can be written:


$$\overset{+}{\underset{g,t}{W}}+\overset{-}{\underset{g,t}{W}}+\overset{+}{\underset{d,t}{W}}+\overset{-}{\underset{d,t}{W}}+\overset{+}{\underset{GRF,t}{W}}+\overset{-}{\underset{GRF,t}{W}}\overset{+}{\underset{inert,t}{+\overset{+}{\underset{SA,t}{W}}+\overset{-}{\underset{SA,t}{W}}=W}}+\overset{-}{\underset{inert,t}{W}}$$ (21)

$$\overset{+}{\underset{g,n}{W}}+\overset{-}{\underset{g,n}{W}}+\overset{+}{\underset{inert,n}{\overset{+}{\underset{SA,n}{W}}+\overset{-}{\underset{SA,n}{W}}=W}}+\overset{-}{\underset{inert,n}{W}}$$ (22)

$$\overset{+}{\underset{g,b}{W}}+\overset{-}{\underset{g,b}{W}}+\overset{+}{\underset{inert,b}{\overset{+}{\underset{SA,b}{W}}+\overset{-}{\underset{SA,b}{W}}=W}}+\overset{-}{\underset{inert,b}{W}}$$ (23)

To evaluate the exploitation of the input positive work, a new parameter, named propulsion efficiency of skiing, was defined:


$$\eta_{S}=\frac{{W_{inert,t}}^{+}+{W_{inert,t}}^{-}}{{W_{g}}^{+}+{W_{SA}}^{+}}$$ (24)

where the resultant inertial mechanical work in the skiing direction (${W_{inert,t}}^{+}+{W_{inert,t}}^{-}$) was taken as the useful “output” mechanical work, and the sum of the positive gravity and $W_{SA}$, as the “input” work.

### Post processing

The calculated data were divided into ski turns. The beginning and end of each ski turn were defined by the intersections of the COM and the MAJ trajectories projected on the ski slope [29]. To obtain average data curves over a ski turn, the data was cubically interpolated for each ski turn to the same number of data points and expressed as a percentage of the ski turn. For each subject, the average ski turn values were calculated across all his ski turns (for three subjects N = 36, for one subject N = 24, and for one subject N = 12). From the average subject data, the overall average curves with standard deviations (SD) were calculated over all the subjects (N = 5).

## Results

**Fig 5:**
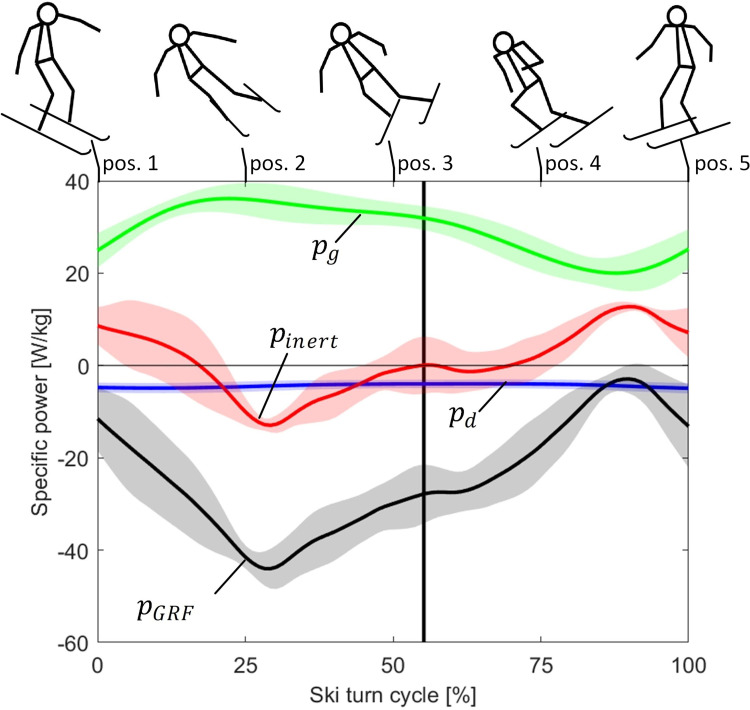
Average specific mechanical powers of the gravity (${𝐩}_{g}$, green), air drag (${𝐩}_{d},$ blue), inertial force (${𝐩}_{inert}$, red) and of the ground reaction force (${𝐩}_{GRF}$, black) at the skier’s COM within a ski turn.The curves represent the mean for five subjects and the shaded area its standard deviation (± SD). The duration of the ski turn is given in percentage of the ski turn cycle. The vertical black line represents the gate position. At the top of the figure relevant stick figures of the skier are shown.

For comparison reasons, power and work were presented as mass specific and annotated with lowercase letters. Fig 5 shows the average specific mechanical powers of the external forces acting on the skier’s COM within a ski turn. The specific gravity power $p_{g}$ was positive over the whole ski turn and propelled the skier down the slope, while the specific air drag power $p_{d}$ and the specific GRF power $p_{GRF}$ were negative and thus resisted the skier’s movement. The power balance of $p_{g}$, $p_{d}$ and $p_{GRF}$ resulted in the specific inertial power $p_{inert}$ (Equation 5). Positive $p_{inert}$ indicates to accelerated skier movement, while negative $p_{inert}$ to decelerated movement.

**Fig 6:**
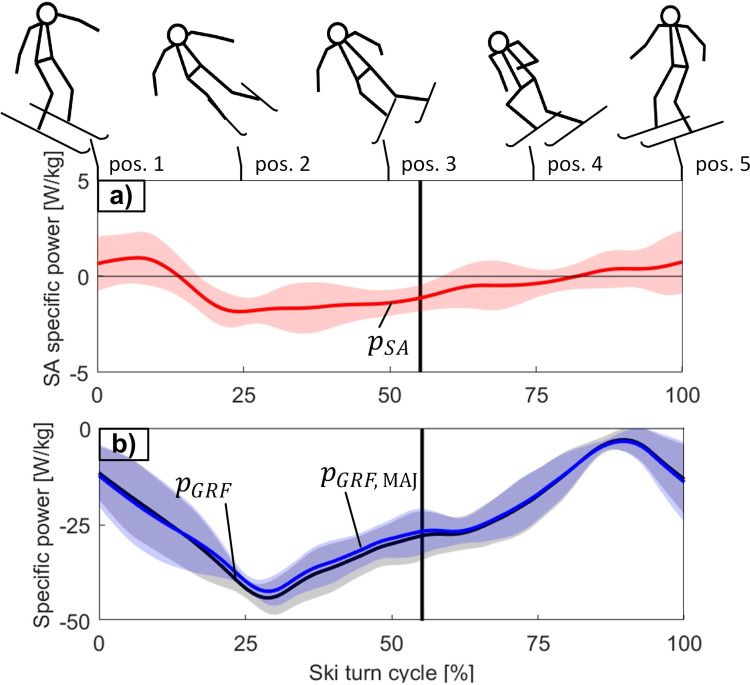
Average specific power resulting from the skier’s activity (${𝐩}_{SA}$, red), including the specific mechanical power resulting from the ground reaction force at the skier’s MAJ (${𝐩}_{GRF,MAJ}$, blue) and at the COM (${𝐩}_{GRF},$ black) within a ski turn. The curves represent the mean for five subjects, and the shaded area its standard deviation (± SD). The duration of the ski turn is given in percentage of the ski turn cycle. The vertical black line represents the gate position. At the top of the figure relevant stick figures of the skier are shown.

Fig 6a shows the average specific power $p_{SA}$ resulting from the skier’s activity within a ski turn, while Fig 6b shows the average specific power of the GRF acting at the skier’s MAJ ($p_{GRF,MAJ}$) and acting at the skier’s COM ($p_{GRF}$). $p_{SA}$ was positive at the beginning and at the end of the ski turn, while in the rest of the ski turn $p_{SA}$ was negative. Positive $p_{SA}$ indicates to skier power generation, negative $p_{SA}$ to skier power absorption.

**Fig 7:**
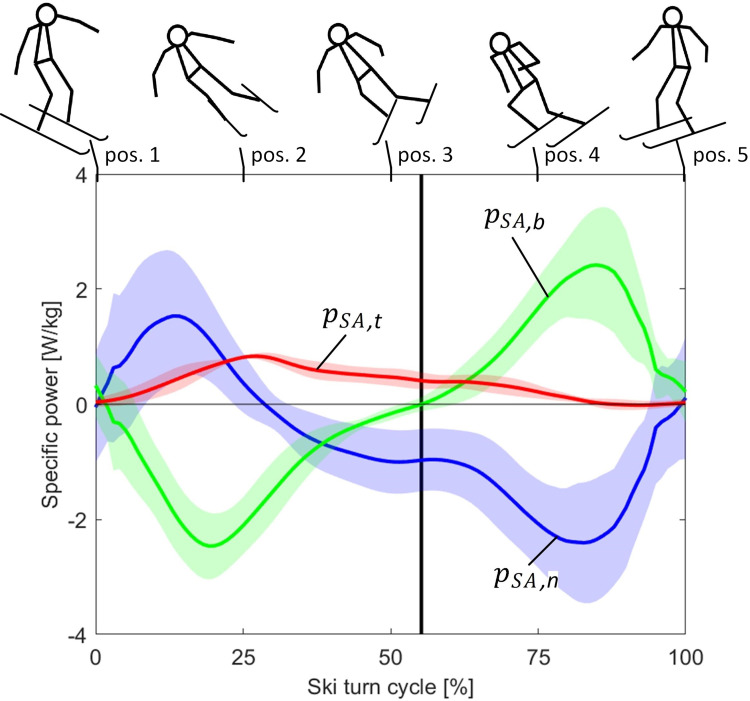
Average specific mechanical power resulting from the skier activity in the tangential (${𝐩}_{SA,t}$, black), normal (${𝐩}_{SA,n}$, blue) and binormal (${𝐩}_{SA,b}$, green) directions of the MAJ trajectory within a ski turn. The curves represent the mean for five subjects, and the shaded area its standard deviation (± SD). The duration of the ski turn is given in percentage of the ski turn cycle. The vertical black line represents the gate position. At the top of the figure relevant stick figures of the skier are shown.

Fig 7 shows the average specific power resulting from the skier’s activity in the tangential ($p_{SA,t}$), normal ($p_{SA,n}$) and binormal ($p_{SA,b}$) directions of the MAJ trajectory within a ski turn. Although $p_{SA}$ oscillated around zero with positive and negative values (Fig 6a), $p_{SA,t}$ was positive almost the whole ski turn, meaning that the skiers produced positive $p_{SA,t}$ even when performing negative $p_{SA}$.

The fundamental skiing kinematics is reflected by $p_{SA,n}$ and $p_{SA,b}$ profiles (Fig 7). Within the ski turn, ${\overset{⇀}{e}}_{n}$ was nearly parallel to the ski slope plane, while ${\overset{⇀}{e}}_{b}$ was nearly perpendicular to the ski slope (Fig 3, pos. 2–4). The skier movements in the direction of ${\overset{⇀}{e}}_{n}$ were mostly influenced by the centrifugal force, while the skier movements in the direction of ${\overset{⇀}{e}}_{b}$ were mostly influenced by the gravity force. At the beginning of the ski turn (Fig 7, pos. 1), the skier was in his upright, “extended” position and $p_{SA,n}$, $p_{SA,b}$ and $p_{SA,t}$ were close to zero. Between positions 1 and 2, the skier leaned towards the ski turn centre, i.e., against the centrifugal force and along the gravity force. Therefore, $p_{SA,n}$ increased, while $p_{SA,b}$ decreased. After position 2, the skier compressed, which is why also $p_{SA,n}$ became negative (Fig 7). $p_{SA,b}$ remained negative until passing the gates. At this point, the skier began to lift his COM against gravity, thus $p_{SA,b}$ became positive and reached its maximum between positions 4 and 5. Simultaneously, $p_{SA,n}$ reached its minimum. From here on, $p_{SA,b}$ decreased and $p_{SA,n}$ increased to zero till the end of the ski turn, when the skier was again in his upright “extended” position (Fig 7, pos. 5).

**Fig 8:**
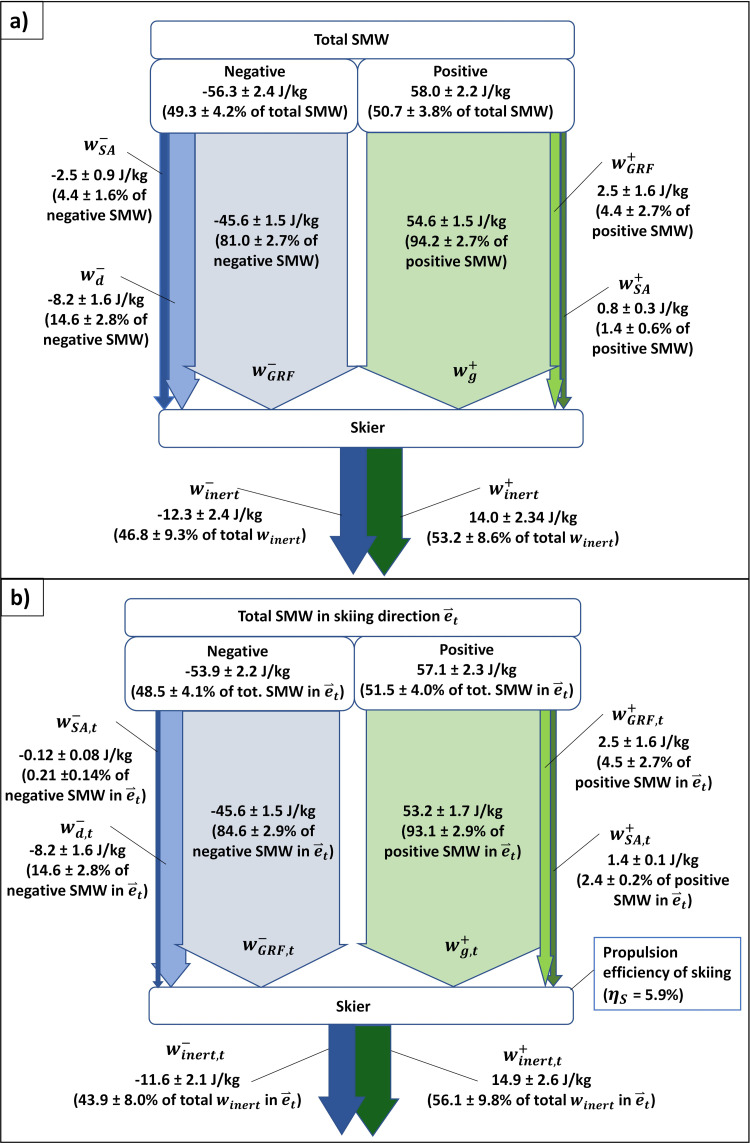
Specific mechanical work (SMW) of the two-point skier model performed over a ski turn. **a)** Total SMW. **b)** SMW performed in the skiing direction. Abbreviations: ${𝐰}_{SA}$ — SMW resulting from the skier activity, ${𝐰}_{d}$ — SMW of the air drag; ${𝐰}_{GRF}$ — SMW of the ground reaction force (GRF); ${𝐰}_{g}$ — SMW of the gravity force; ${𝐰}_{inert}$ — SMW of the inertial force. *t* index refers for the work performed in the tangential direction of the trajectory of the midpoint between the ankle joints (MAJ), i.e., in the skiing direction. Plus (“+”) and minus (“-“) signs refer to positive and negative mechanical work.

In Fig 8a the average total specific mechanical work (SMW) performed by the 2-point skier model over a ski turn is presented in an arrow chart. The total positive SMW was slightly larger than the total negative SMW (1.4 percentage points). The total positive SMW consisted of the work performed by the gravity ($\overset{+}{\underset{g}{w}}$, 94.2%), of the work performed by the GRFs ($\overset{+}{\underset{GRF}{w}}$, 4.4%) and of the SAMW ($\overset{+}{\underset{SA}{w}}$, 1.4%), while the total negative SMW consisted of the work dissipated against air drag ($\overset{-}{\underset{d}{w}}$, 14.6%), of the work performed by the GRFs ($\overset{-}{\underset{GRF}{w}}$, 81.0%) and of the work absorbed in the skiers’ bodies ($\overset{-}{\underset{SA}{w}}$, 4.4%). $w_{d}$ was exclusively negative, while $w_{g}$ was exclusively positive. The “resultant” inertial specific mechanical work $w_{inert}$ was positive and negative, indicating to accelerating and decelerating movement of the skiers, where $\overset{+}{\underset{inert}{w}}$ was 6.4 percentage points larger than $\overset{-}{\underset{inert}{w}}$. Concerning $w_{SA}$, the skiers produced 3.1 times more negative $w_{SA}$ than positive.

In Fig 8b the SMW performed in the tangential direction of the MAJ trajectory (i.e., in the skiing direction) is presented. In brackets, the SMW portions relative to the corresponding total SMW in the tangential direction are given. The negative and positive SMW performed in the skiing direction presented almost all of the total negative and total positive SMW (Fig 8a), 91.7% and 95.7% respectively. $\overset{+}{\underset{g,t}{w}}$ presented 97.4% of $\overset{+}{\underset{g}{w}}$, $\overset{+}{\underset{GRF,t}{w}}$ presented 100.0% of $\overset{+}{\underset{GRF}{w}}$, while $\overset{+}{\underset{SA,t}{w}}$ was even greater than $\overset{+}{\underset{SA}{w}}$ by 71.1% at the expense of negative $w_{SA}$ performed in other directions. Also, $\overset{+}{\underset{inert,t}{w}}$ was 6.4% greater than $\overset{+}{\underset{inert}{w}}$. The average propulsion efficiency of skiing $\eta_{S}$ was 5.9%, which indicated to accelerating movement at the observed ski turns. Also, the work balance of the two-point skier model implied in the Equations 20–23 was confirmed.

## Discussion

The main findings of the study are summarized here. A detailed discussion follows.

(a) Skiers performed 3.1 times more negative $w_{SA}$ than positive (Fig 8a), which answers an important question about the dominant muscle activity in alpine skiing [30].
(b) Although $w_{SA}$ was mainly negative (Fig 8a), $w_{SA,t}$ was exclusively positive (Fig 8b) on the expense of negative $p_{SA}$ performed in normal and binormal skiing directions (Fig 7). Positive $w_{SA,t}$ and its power are a characteristic of ski turn mechanics and only partly reflect the skier’s own propulsion.
(c) Positive $w_{SA}$ in the skiing direction was small compared to the gravity positive work (2.4% vs. 93.1%, Fig 8b). 4.5% of the total positive mechanical work in the skiing direction represented positive GRF work (Fig 8b), but its credibility is questioned (see section *Overestimation of air drag may lead to positive GRF work*). Regarding energy dissipation, 81.0% of the total negative SMW was dissipated in the ski-snow contact, 14.6% was dissipated against the air drag and 4.4% was absorbed in the skiers’ bodies (Fig 8a).

### Skier activity mechanical power and work

Skiers generate/absorb $w_{SA}$ by extending and compressing their bodies, i.e., by moving their COM relative to the skis. Researchers [15–19] proposed the pumping mechanism as a source for propelling in alpine skiing, similar to pumping on a swing [31,32], but authors of the present study stay reserved about this interpretation.

The skier activity power in the skiing direction is a product of $F_{GRF,t}$ and $v_{SA,t}$. $F_{GRF,t}$ as a rule, acts in the opposite direction of skiing and is thus negative, while $v_{SA,t}$ is calculated as the difference between $v_{COM,t}$ and $v_{MAJ,t}$. Since the MAJ trajectory is normally longer than the COM trajectory (Fig 3), $v_{MAJ,t}$ is greater or equal to $v_{COM,t}$ (being equal only when the skier is in his upright position), which makes $v_{SA,t}$ negative or zero. $p_{SA,t}$ can therefore only be positive or zero (Fig 7). Note, that if there was no friction between the skis and the snow, $F_{GRF,t}\;$and consequentially also $P_{SA,t}$ would be zero. Therefore, friction is essential for positive $P_{SA,t}$.

Since $w_{SA}$ is determined by the skier activity power, the most eloquent are the power profiles of $p_{SA}$ (Fig 6a) and of the skier’s activity power in the individual directions of the skiing trajectory (Fig 7). According to Equation 9, the skier activity power in the skiing direction can be expressed as $p_{SA,t}={p_{SA}-p}_{SA,n}-p_{SA,b}$. Here, despite negative $p_{SA}$, positive $p_{SA,t}$ can be obtained in case of sufficiently negative $p_{SA,n}$ and $p_{SA,b}$. Since $p_{SA,n}$ and $p_{SA,b}$ were on average more negative than positive, the average positive $w_{SA,t}$ presented even 171.1 ± 15.8% of the total positive $w_{SA}$ (Fig 8).

It seems that the role of the skier’s activity is, in addition to minimizing energy dissipation and choosing the best track [18], also to enable such a movement that allows an increase in $p_{SA,t}$ at the expense of a decrease in $p_{SA,n}$ and $p_{SA,b}$. Such skier’s activity transforms the energy from one direction into another. It seems that for obtaining positive $p_{SA,t}$, the magnitude of $p_{SA}$ is of secondary importance as an energy source, and that more important is the distribution of $p_{SA}$ over $p_{SA,n}$ and $p_{SA,b}$. Moreover, it can be assumed, that the amount of positive $p_{SA,t}$ is generally more influenced by the ski track than by the skier’s activity and is thus, above all a characteristic of the ski turn mechanics. In that manner, skiers can produce more positive $p_{SA,t}$ in slalom than in downhill and giant slalom.

### SAMW in relation to muscle work

The SAMW is performed by the skier’s muscles. The true muscle work is difficult to measure due to the possible muscle coactivations [33] and due to the sensitivity of the musculoskeletal model to the accuracy of the muscle-tendon attachments to the bones. Hence, muscle work and SAMW are not the same, but proportionally dependant. To perform positive SAMW, primarily concentric muscle contractions, which produce positive muscle work, are required, whereas for performance of negative SAMW primarily eccentric muscle contractions, which produce negative muscle work, are needed.

The scientific community is uncertain which type of muscle work dominates in alpine skiing [30]. Kröll [13] and Berg [14] found that eccentric muscle activity of the quadriceps muscle is dominant and attributed this to the higher negative mechanical work performed when descending, similar to downhill walking [33]. On the other hand, Petrone [34] found no pronounced dominance of eccentric over concentric muscle contractions in the quadriceps muscles during racing, training and recreational slalom.

Till now, only Meyer [11] has evaluated SAMW for the skier’s COM movements relative to the skis. He reported that the average $w_{SA}$ at the end of a ski turn was close to zero, meaning that skiers produced almost equal amounts of positive and negative $w_{SA}$. The present study challenges these findings, since a significant dominance of negative $w_{SA}$ over positive was found (Fig 8a). This finding gives basis for confirmation of the dominating eccentric muscle activity in skiing and presents important information especially for the training of skiers [35].

### Overestimation of air drag may lead to positive GRF work

Although the GRF work is generally negative, positive GRF work of up to 3% of total positive work was found in giant slalom [11] and slalom [36,37]. Also in the present study, positive GRF work accounted for 4.5% of the total positive SMW in the skiing direction (Fig 8b) but is questioned.

When observing a gliding ski alone, its GRF can only be resistive to its movement [38]. When observing a skier gliding on two skis, the resulting GRF could in theory also act propulsive to the skier’s movement, e.g., when skating push-offs or pole push-offs are used. Even then, at high velocities, it is more likely that with skier’s propulsive activity only reduction of the negative $w_{GRF}$ is achieved, rather than positive $w_{GRF}$.

It must be emphasized, that positive $w_{GRF}$ is very sensitive to air drag. Since the skier’s movement is generally resisted by the GRF and the air drag, overestimation of the air drag decreases the resistant GRF, which may at some moments lead even to falsely propulsive GRF work.

### Propulsion efficiency of skiing

Mechanical work efficiencies in biomechanics are usually defined in relation to the total metabolic cost [33,38,39]. In the present study, propulsion efficiency in skiing $\eta_{S}$ was defined as a measure of utilization of the total input mechanical work (Equation 24). In case the skier descends with a constant velocity, $\eta_{S}$ is zero, in case of an accelerating skier movement, $\eta_{S}$ is positive, and in case of a decelerating skier movement, $\eta_{S}$ is negative. This efficiency may become an important parameter for evaluation of the skiing quality of individual ski turns or ski course sections.

### Comparison to other sports

Even though the SAMW has been normalized by the skier’s mass, it cannot be directly compared with the mechanical work performed in other sports, if the duration of the compared activity is not the same. However, $p_{SA}$ can be compared to the specific external power performed in other sports (usually notated as $p_{ext}$). The absolute $p_{SA}$ was on average 0.90 ± 0.55 W·kg^-1^, which is somewhat lower than $p_{ext}$ performed in other sports; e.g., in running at 3.08 m·s^- 1^, $p_{ext}$ was reported to be 3.9 W·kg^-1^ [2], while in sprint running $p_{ext}$ was much higher, ranging from approximately 10 W·kg^-1^ to 20 W·kg^-1^ at sprinting velocities between 5.0 m·s^-1^ and 6.5 m·s^-1^ [40]. The lower mechanical power production in alpine skiing is suspected to be due to near-isometric muscle contractions, due to a higher number of muscle coactivations [14,41] and due to lower metabolic effort of the skiers.

### Limitations and future work

The limitation of the air drag model accuracy and its influence on the GRF work was discussed in the section “*Overestimation of air drag may lead to positive GRF work*”. Other errors may arise from motion capture accuracy, sensor alignment, synchronization procedures, skier’s COM estimation and from data processing (filtering, differentiation). However, by averaging the results over multiple ski turns, the influence of these errors was minimized.

Another source of error may be the usage of the MAJ instead of the actual GRF application point. The error due to this modelling simplification is expected to be negligible, as the structures between MAJ and the actual GRF application point are relatively stiff and therefore contribute minimally to energy generation or absorption.

Since the skier is clamped to the skis, he can also move his COM forth and back with respect to the MAJ and thus produce rotational skier activity mechanical work. However, athletes naturally seek minimal torque loading, therefore the corresponding work is mostly negligible in human locomotion [38]. Also, previous studies on rotational kinetic energy in alpine skiing [20] found this energy negligible in comparison to other energies. Therefore, the authors believe that neglecting rotational skier activity mechanical work is acceptable. However, to consider this work, a more complex skier model would be needed, where also GRFs and torques should be measured and applied on the model.

Due to the close gate setup, the skiing speed in the present study was relatively low (11.5 ± 1.1 m·s^-1^) compared to typical giant slalom racing conditions. At higher speeds, under different gate setups, slope inclinations, and levels of skier experience, different results may be observed, which would be of great interest for future investigations.

Another part of the SAMW is the work required to move the skier’s segments relative to the COM, which is also to be considered in future studies. For a more accurate evaluation of the mechanical work performed in alpine skiing, it is also suggested to measure the GRFs on the skis and poles in addition to the skier’s kinematics. This way, the SAMW could be verified using a different method [42] and the GRF work could be determined more precisely.

## Conclusions

A novel two-point model of the skier was introduced to evaluate mechanical power and work in giant slalom. The skier activity power profiles within a ski turn were represented in the normal, binormal and tangential directions of the skiing trajectory. The positive skier activity mechanical work $W_{SA}$ performed in the skiing direction over a ski turn presented 2.4% of the total positive work in the skiing direction. Negative $W_{SA}$ was 3.1 times larger than the positive, which answers an important question about the dominating muscle activity in alpine skiing [30]. This is particularly important information for skier training. Although $W_{SA}$ was mostly negative, $W_{SA}$ in the skiing direction was positive over the whole ski turn. Positive $W_{SA}$ was found to be mostly a consequence of sufficiently negative skier activity power/work performed in other skiing directions. Moreover, a novel parameter for skiing efficiency evaluation was proposed. Understanding of the mechanisms underlying skier propulsion during a ski turn are still not fully understood, therefore further research involving mathematical skier models is suggested.
